# Type and timing of adverse childhood experiences differentially affect severity of PTSD, dissociative and depressive symptoms in adult inpatients

**DOI:** 10.1186/s12888-016-1004-5

**Published:** 2016-08-19

**Authors:** Inga Schalinski, Martin H. Teicher, Daniel Nischk, Eva Hinderer, Oliver Müller, Brigitte Rockstroh

**Affiliations:** 1 Department of Psychology, University of Konstanz, P.O. Box 905, Konstanz, 78457 Germany; 2 Department of Psychiatry, Harvard Medical School, Boston, MA USA; 3 Developmental Biopsychiatry Research Program, McLean Hospital, Belmont, USA; 4 Center for Psychiatry, Reichenau, Germany

**Keywords:** Childhood adversities, Adult psychopathology, Shutdown dissociation, Depression, PTSD, Dose-dependent, Type and timing

## Abstract

**Background:**

A dose-dependent effect of Adverse Childhood Experiences (ACE) on the course and severity of psychiatric disorders has been frequently reported. Recent evidence indicates additional impact of type and timing of distinct ACE on symptom severity experienced in adulthood, in support of stress-sensitive periods in (brain) development. The present study seeks to clarify the impact of ACE on symptoms that are often comorbid across various diagnostic groups: symptoms of posttraumatic stress disorder (PTSD), shutdown dissociation and depression. A key aim was to determine and compare the importance of dose-dependent versus type and timing specific prediction of ACE on symptom levels.

**Methods:**

Exposure to ten types of maltreatment up to age 18 were retrospectively assessed in *N* = 129 psychiatric inpatients using the Maltreatment and Abuse Chronology of Exposure (MACE). Symptoms of PTSD, shutdown dissociation, and depression were related to type and timing of ACE. The predictive power of peak types and timings was compared to that of global MACE measures of duration, multiplicity and overall severity.

**Results:**

A dose-dependent effect (MACE duration, multiplicity and overall severity) on severity of all symptoms confirmed earlier findings. Conditioned random forest regression verified that PTSD symptoms were best predicted by overall ACE severity, whereas type and timing specific effects showed stronger prediction for symptoms of dissociation and depression. In particular, physical neglect at age 5 and emotional neglect at ages 4–5 were related to increased symptoms of dissociation, whereas the emotional neglect at age 8–9 enhanced symptoms of depression.

**Conclusion:**

In support of the sensitive period of exposure model, present results indicate augmented vulnerability by type x timing of ACE, in particular emphasizing pre-school (age 4–5) and pre-adolescent (8–9) periods as sensitive for the impact of physical and emotional neglect. PTSD, the most severe stress-related disorder, varies with the amount of adverse experiences irrespective of age of experience. Considering type and timing of ACE improves understanding of vulnerability, and should inform diagnostics of psychopathology like PTSD, dissociation and depression in adult psychiatric patients.

## Background

Adverse childhood experiences (ACE) shape the individual vulnerability and often precede psychopathology within and across diagnostic boundaries [1, 2]. Current models on etiology describe symptom severities mainly as a linear function of cumulative ACE, e.g. [2–6]. Besides the dose-dependent effect, recent evidence indicates a distinctive impact of type and timing of ACE on symptom severity experienced in adulthood, supporting the notion of stress-sensitive periods in (brain) development in childhood [7, 8]. The sensitive type and timing model offers an alternative (and complementary) explanation for dose-dependent effects, because alongside with the exposure to a variety of ACE, the likelihood of exposure to a specific type of ACE in a critical period increases as well. Both models (dose-dependent and sensitive type and timing) have been supported by empirical data, however not yet sufficiently contrasted in their predictive power for symptom dimensions shown to increase in intensity and frequency in response to adversities and trauma: symptoms of posttraumatic stress disorder (PTSD), dissociation and depression. These symptoms were chosen as prominent and comorbid in different diagnostic categories [9–11].

Cumulative or dose-dependent effects of environmental adversities have been quantified by trauma load, ACE load (multiplicity or number of event types; see [3, 6, 12]) or overall severity of exposure [13]. These measures are strongly interrelated [14] and assess the global level of exposure rather than the frequency of distinct adversities or traumatic event types. For instance, symptoms of PTSD have been reported to increase in a dose-dependent manner e.g., linked to the different types of traumatic experiences in a war and in displacement affected population [3] as well as to the severity of childhood adversities in a sample of German psychiatric inpatients [5]. Similarly, dose-dependent effects have been reported for symptoms of dissociation and depression in these patients [4, 5, 15, 16].

Moreover, the augmenting impact of certain types of ACE experienced at certain times during development ask for a broader perspective beyond an exclusive dose-dependent modulation. In particular sexual and physical abuse [17, 18], or emotional neglect and abuse [19] have been shown to increase levels of PTSD symptoms in adulthood. Further, more severe PTSD has been reported for individuals who were exposed to ACE at 3–5 years of age [20]. Other results suggest higher vulnerability to PTSD for exposure beyond the age 11 [21]. Dissociative symptoms (particularly the ongoing shutting down of sensory, motor and speech systems together with emotional overregulation) have been proposed to emerge in susceptible individuals as a defensive response from past highly proximal traumatic stress such as sexual abuse [22, 23]. This relation between sexual abuse and physical maltreatment in childhood and shutdown dissociation was confirmed in adult psychiatric inpatients with Borderline Personality Disorder and depression [15, 24]. Similarly, amplified symptoms of shutdown dissociation were related to different types of emotional neglect as well as non-verbal emotional abuse, and peer emotional abuse with peak vulnerability at age 13–14 in psychiatric patients with psychotic disorders [25]. More intense depressive symptoms have been reported to vary with exposure to emotional abuse and neglect [19, 26], or sexual and physical abuse [27, 28].

While results indicate that particularly ACE exposure before age 12 may predispose for the later development and course of depression [21, 29, 30], Khan and colleagues conclude from their detailed analysis of sensitive periods in a community sample (with a portion of 33 % of individuals with lifetime depression and 4 % of current clinical depression) a specific type and timing effect. More precisely, non-verbal emotional abuse in males and peer emotional abuse in women each at age 14 increased the risk for later diagnosis of depression as well as for current depressive symptoms [31].

While current results suggest vulnerable time windows particularly for sexual abuse, loss of a parent and traumatic experiences [7, 21, 29], understanding whether this sensitivity is type and timing-specific in their interaction with the vulnerability to mental illness requires a more comprehensive assessment of the various ACE in fine-grained developmental periods. Moreover, it is necessary to compare the predictive power of type and timing in relation to global parameters (multiplicity of ACE, overall severity of ACE) for symptoms of PTSD, dissociation and depression across different diagnostic groups. While both, a dose-dependent and a sensitive-period influence of environmental adversities have been examined in community samples and in clinical samples e.g., [3, 5], the present study directly compared both hypotheses within the same, larger sample of psychiatric inpatients. This transdiagnostic approach is in line with the proposed model for trauma-related psychopathology of the Research Domain Criteria by the National Institute of Mental Health, that suggests a link between psychopathology and abnormal neurobiology across disorders [32, 33].

In the present study a larger sample of inpatients with different mental disorders were comprehensively assessed for ACE and symptoms of PTSD, dissociation and depression (irrespective of their primary diagnosis). Our main objectives were (1) to replicate the relationship between exposure to global scores (duration, multiplicity and overall severity) as well as trauma load in childhood and adulthood, and (2) to compare the predictive power of type and timing to the predictive power of global scores (duration, multiplicity, overall severity)—both derived from the Maltreatment and Abuse Chronology of Exposure (MACE) Scale [14]. Moreover, we sought to establish the direction of strongest predictors using the best fitting linear regression model for each symptom dimension.

## Method

### Ethics statement

Prior to the assessment each participant provided written informed consent. All patients were in post-acute state and capable of participation as confirmed by the responsible psychologist or psychiatrists. The study was reviewed and approved by the Institutional Review Board (Ethics Committee) of the University of Konstanz.

### Participants and setting

One hundred twenty nine adult inpatients of different diagnostic groups (41.9 % female) were recruited at the local Center for Psychiatry. Participants were on average *M* = 26.1 years (*SD* = 5.5), and had been admitted on average *M* = 1.9 (*SD* = 1.3) times to a psychiatric hospital (see Table 1 for demographic and clinical information). All patients had a diagnosis of a least one of the following ICD-10 diagnostic categories [34]: F1-F19 (mental and behavioral disorders due to psychoactive substance use), F2-F29 (psychotic spectrum disorders), F30-F39 (affective disorders), F40-F48 (neurotic, stress-related and somatoform disorders), F50-F59 (behavioral syndromes associated with physiological disturbances and physical factors) and F60-F69 (disorders of adult personality and behavior). All patients participated in the hospital’s standard treatment protocols, which include medication, group therapy, physical exercise and adjunct cognitive behavioral psychotherapy. Nineteen of the 129 patients were unmedicated and 110 patients were on stable medication regimen of at least 2 weeks prior to data assessment, *n* = 50 patients receiving antidepressants and *n* = 82 typical and atypical neuroleptics. Of the 110 patients *n* = 88 were treated with either antidepressants (*n* = 28) or neuroleptics (*n* = 60), and *n* = 22 received a combination of neuroleptics and antidepressants. None of the patient took benzodiazepines at the time of assessment. In patients treated with neuroleptics (*n* = 82) the mean chlorpromazine equivalent dose (CPZ) was *M* = 410.1 (*SD* = 363.9), which is close to the commonly recommended maintenance dosage.

**Table 1:** Sample characteristics: demographic data and diagnostic categories according to the ICD-10

	*M* (*n*)	*SD* (%)
Demographic Data		
Female sex (*n*, %)	54	41.9 %
Age (in years)	26.1	5.5
Number of Hospitalizations	1.9	1.3
Education (in years)	11.4	1.8
University entrance diploma (*n*, %)	57	44.2 %
Clinical Characteristics		
ICD-10: F1-F19: Mental and behavioral disorders due to psychoactive substance use	46	35.7 %
ICD-10: F2-F29: Psychotic spectrum disorders	63	48.8 %
ICD-10: F30-F39: Affective Disorders	54	41.9 %
ICD-10: F40-F48: Neurotic, stress-related and somatoform disorders	42	32.6 %
ICD-10: F50-F59: Behavioral syndromes associated with physiological disturbances and physical factors	4	3.1 %
ICD-10: F60-F69: Disorders of adult personality and behavior	30	23.8 %
Number of Different Diagnosis Across Categories (*M*, *SD*)	1.9	0.7
Single Diagnosis (*n*, %)	41	31.8 %
One Comorbid Disorders (*n*, %)	66	51.2 %
Medication		
Antidepressants (*n*, %)	50	38.8 %
Atypical Neuroleptics (*n*, %)	82	63.6 %
Symptom Severities		
Shutdown Dissociation (*M*, *SD*)	6.8	6.0
PTSD Symptom Severity (*M*, *SD*)	7.9	11.5
Depression Symptom Severity (*M*, *SD*)	15.9	7.4

*ICD*-*10* International Classification of Diseases (ICD), 10th Version

### Measures and instruments

*Childhood adversities* were assessed by interview using the MACE Scale [14, 24]. The MACE captures the exposure to ten types of ACE (parental physical and verbal abuse, parental non-verbal emotional abuse, familial and non-familial sexual abuse, witnessed physical violence towards parents, witnessing violence towards siblings, peer emotional and peer physical violence, emotional and physical neglect). The scale shows excellent test-retest reliability and convergent validity to the childhood trauma questionnaire [13, 14, 24]. For each of the 75 items binary responses were recorded and for positive responses the age of occurrence specified for every single year of life up to age 18. The raw data were used to define type and timing specific scores as well as global measures of ACE exposure. ACE are globally quantified by (a) the MACE MULTI score indicating the number of different types of childhood adversities that fulfilled a defined threshold of severity and the MACE MULTI ranges from 0 to 10 (cut-offs were retrieved from [24]), and (b) the MACE SUM score indicating overall severity of exposure ranging from 0 to 100 [14]. (c) The global measure MACE duration is quantified by the mean cross sum of the MACE severity scores along the 18 years, therefore scores ranging from 0 to 100. (d) Severity scores of each type and timing as well as global ACE measures are considered in random forest regression.

In addition, traumatic experiences were assessed with the event checklist [35] using the sum of different types of events as index of trauma load. Sum scores were calculated separately for traumatic event types that were experienced up to age 18 (trauma load in childhood) and those reported after the 18th birthday (trauma load in adulthood).

*Symptom severity* was determined for dissociative responding using the 13-item Shutdown Dissociation Scale (Shut-D) [22, 23] in a structured interview. The Shut-D allows for the comprehensive assessment of the severity of dissociative experiences during the past 6 months. Following the guidelines of administering and analyzing the Shut-D, reports affected by side effects of medication or dissociative symptoms due to alcohol or other drugs were excluded [22]. The frequency of dissociative experiences was quantified on a scale between 0 (not at all), 1 (once a week or less), 2 (2–4 times a week), to 3 (5 or more times a week). The sum score (ranging from 0 to 39) serves as an indicator for the severity of shutdown dissociation. The scale showed excellent internal reliability as well as test-retest reliability, high convergent validity, and satisfactory predictive and discriminatory validity [22].

PTSD symptom severity was defined by the sum score of the PTSD Symptom Scale-Interview (PSS-I), which follows the criteria based on the Diagnostic and Statistical Manual of Mental Disorders, Fourth Edition (DSM-IV) [36]. The PSSI has high reliability and validity and is comparable to more complex instruments for quantifying PTSD [37].

The severity of depression was determined by expert ratings on the 21-item Hamilton Depression Scale that were based on information reported by the patient, observations during the interview and observations made by health care staff [38]. The scale has been extensively used to obtain severity scores for depression and is well established in clinical research [39].

### Statistical analysis

Analyses were performed using R version 2.15.1 and SPSS 20.0. Per hypothesis (1) the impact of ACE multiplicity (or the dose-dependent effect) on symptom severities was examined for each symptom dimension (PTSD, shutdown dissociation and depression) by univariate analyses of variance (ANOVA) with the between-subjects factor multiplicity, comparing subgroups according to 5 different levels of multiplicity (0 = no childhood adversities (*n* = 13), 1 = one type of MACE MULTI (*n* = 29), 2 = two types of MACE MULTI (*n* = 20), 3 = three types of MACE MULTI (*n* = 18) and 4 = four to ten types of MACE MULTI (*n* = 49; see [12], for a similar approach). In addition, the relationship between the symptom severities and global measures of the MACE duration, multiplicity (MACE MULTI), overall severity (MACE SUM), and the trauma load in childhood and adulthood were evaluated by Pearson correlations. Similarly, correlations determined the relationship between symptom severity and the severity score of each type of ACE. In order to correct for multiple testing the Benjamini-Hochberg procedure was applied [40], resulting in a corrected significance threshold of *α* = 4.3 % for hypothesis 1 and *α* = 3.6 % for the hypothesis 2.

#### Conditioned random forest regression

In fine-grained conditioned random forest regressions predictive modeling is accomplished using the script provided by Teicher and Parigger in the Supplement [14]. In contrast to conventional linear modeling, the conditioned forest regression considers the high co-linearity of reported exposure of types at adjacent ages and does not require specific distribution assumptions [41]. Furthermore, this approach can handle a very large number of predictors modeling the outcome [41], and has been applied in previous studies [8, 25, 31]. We applied a variant of Breiman’s approach with conditionals trees using the R packages ‘party’ [42] and ‘caret’ [43]. This approach rectifies a possible challenge with random forest regression that can inflate predictor importance with high versus low levels of categories. Conditioned random forest models provide an unbiased estimation of predictor importance independent of number of categories, mean values, range, and variance of the predictors [42]. Model training and testing were accomplished using a 10 × 10 Monte Carlo cross validation algorithm [43]. For this, the data set was randomly split into a subsample to train the model (75 % of the data) and a test subset (25 %), therefore providing estimates of the models’ predictive accuracy. Ten runs of the cross validation procedure were performed and model fit, relative variable importance as well as their confidence limits retrieved. The analyses excluded ages or age ranges for types of maltreatment that were reported by less than 5 % of the entire sample. Applying the criterion of none-to low frequent exposure excluded the following types and timings from further analyses: for sexual abuse ages 1–11 and age 15; for physical abuse, parental verbal abuse and witnessed violence to siblings ages 1, 2 and 3 and for interparental violence ages 1–4 were excluded from all random forest regression analysis. For the peer-related types none-to low variation was evident for peer emotional abuse ages 1–4 and for peer committed physical violence ages 1–5. Testing hypothesis (2) relative importance values for each predictor were derived to examine the ‘Sensitivity by Type and Timing’ using severity scores of each MACE type and timing versus global MACE measures on symptom severity. We controlled for the potential impact of gender, trauma load (in childhood and adulthood), diagnostic category (F1, F2 and F6), neuroleptic and antidepressant mediation (as binary variables), as well as number of hospitalizations^1^. The null hypothesis of no difference in relative importance of global predictors compared to the relative importance of a specific type and timing at one or two adjacent ages should be rejected whenever either one model significantly differed in terms of the relative importance. The impact of time of exposure independent of type of exposure was displayed in a graph for easier visualization. Moreover, the type versus global model showed relative importance for type effects regardless of age of exposure as well as global parameters MACE duration, MULTI and SUM. The post-hoc linear modeling evaluated the direction and strength of each predictor. Only those predictors that showed predictive strength derived from the ‘Sensitivity by Type and Timing’ model were entered and the best predicting model was selected using the Akaike information criterion (AIC) [44]. Due to high co-linearity of severity of exposure scores at adjacent ages only the age with the maximal importance was considered in linear regression. Moreover, the high correlation between neuroleptic medication and diagnosis of psychotic spectrum precluded their consideration in linear modeling.

## Results

### Prevalence of adverse childhood experiences per type and timing of exposure

**Fig. 1:**
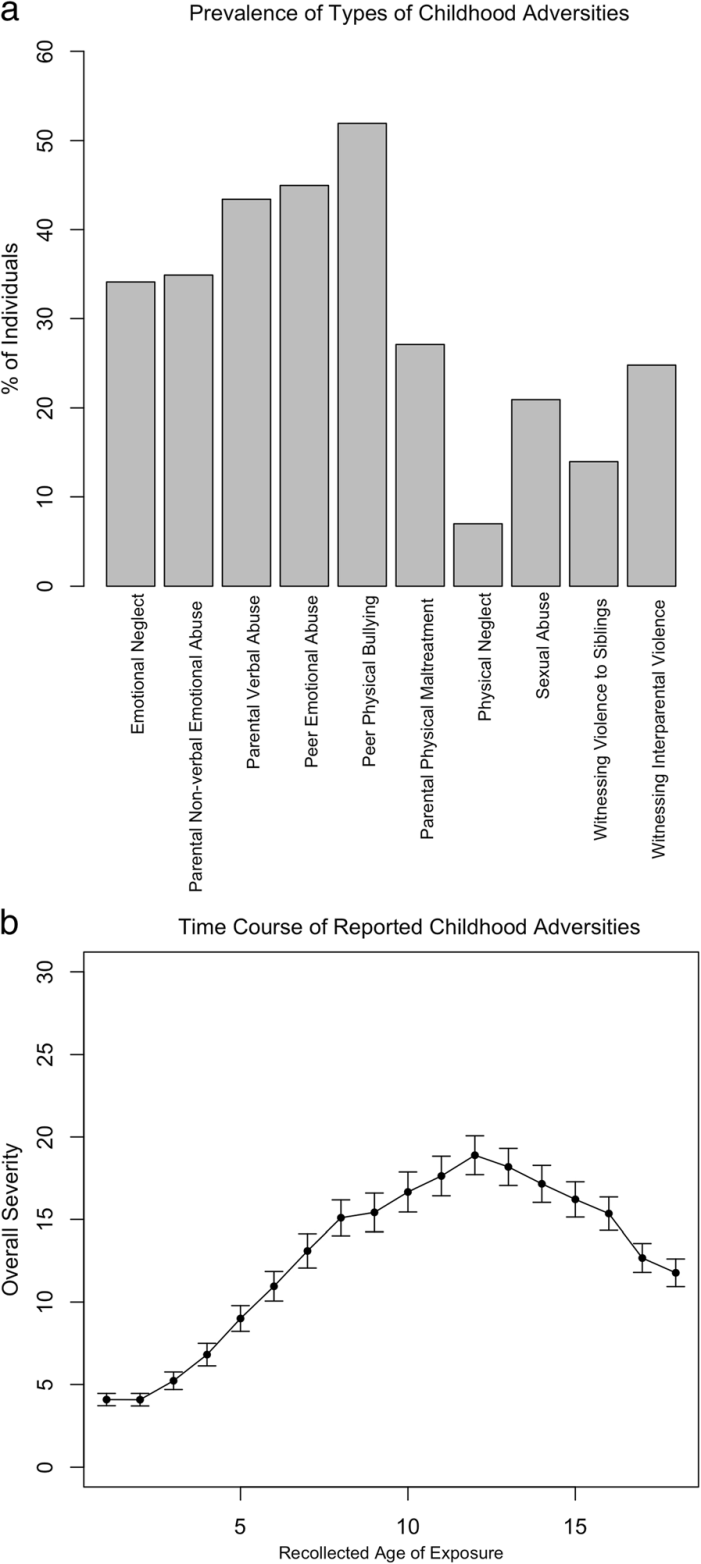
**a** Prevalence (in %) of occurrence per type, **b** Levels of overall severity of childhood adversities (MACE SUM) for the reported age of exposure. Error bars represent the standard deviation of the mean

From the sample of 129 inpatients, altogether 89.8 % met the criteria for one or more ACE types. This prevalence rate exceeds the one reported for healthy populations e.g., 50 % [45] and confirms the high childhood stress load in patients with mental illness. Twenty-three percent were exposed to one type, and around 30 % to 2–3 different types and 37 % to 4–9 different types. On average, individuals reported having been exposed to *M* = 3.0 (*SD* = 2.2) different types of childhood adversities. Regardless of time of exposure, the highest prevalence was found for peer physical abuse (53.2 %), followed by peer emotional abuse (46 %), and parental verbal abuse (43.7 %; Fig. 1a). Considering the timing, the highest peak of overall ACE severity (MACE SUM) and multiplicity (MACE MULTI) was observed at age 13 (Fig. 1b).

### Dose-dependent effects for symptoms of PTSD, shutdown dissociation and depression (Hypothesis 1)

**Fig. 2:**
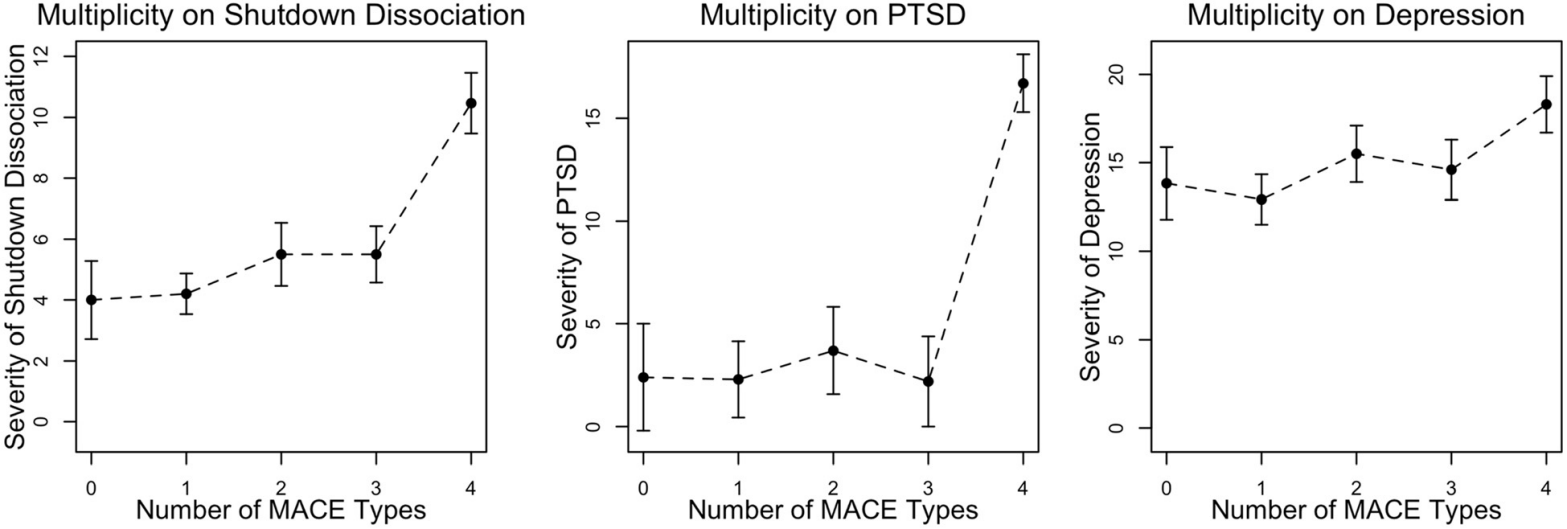
Multiplicity effects for the grouped MACE MULTI scores (0 = no exposure, 1 = exposure to one type, 2 = exposure to two types, 3 = exposure to three types, 4 = exposure to 4 or more types of childhood adversities) for Symptom Severities of PTSD, Shutdown Dissociation and Depression

Comparing participants with MACE MULTI scores of 0, 1, 2, 3 and of 4 or higher showed more severe PTSD symptoms in those participants with ≥ 4 compared to those < 4 events, verifying a significant multiplicity effect (*F*_(4124)_ = 16.13, *p* < .001, *η2* = .34). Similarly, the number of types of the MACE MULTI scores varied with the severity of shutdown dissociation, (*F*_(4124)_ = 8.93, *p* < .001, *η2* = .22) and depression (*F*_(4124)_ = 3.65, *p* = .008, *η2* = .11; see Fig. 2). The MACE overall severity was related to symptoms of PTSD (*r* = .58, *p* < .001, 95 % CI[.44, .68]), symptoms of shutdown dissociation (*r* = .39, *p* < .001, 95 % CI[.27, .53]) and symptoms of depression (*r* = .39, *p* < .001, 95 % CI[.23, .53]).

Further, a longer duration of exposure was related to higher symptom severities (Table 2). Type-specific correlations with symptom severities in Table 2 further confirm substantial relationships between sexual abuse and PTSD symptom severity, emotional neglect and both shutdown dissociation and symptoms of depression.

**Table 2:** Descriptive statistics and correlations between measures of childhood adversity, trauma load and psychopathology (symptoms of PTSD, shutdown dissociation and depression)

Measures of childhood adversities	*M* (*SD*)	PTSD symptom severity	Shutdown dissociation symptom severity	Depression symptom severity
MACE Global Measures				
MACE Duration	12.7 (8.6)	.55, *p* < .001 *, 95 % CI[.41, .66]	.40, *p* < .001 *, 95 % CI[.25, .54]	.46, *p* < .001 *, 95 % CI[.31, .58]
MACE MULTI Multiplicity	3.0 (2.2)	.58, *p* < .001 *, 95 % CI[.43, .70]	.41, *p* < .001 *, 95 % CI[.27, .56]	.37, *p* < .001 *, 95 % CI[.21, .51]
MACE SUM Overall Severity	31.6 (15.6)	.58, *p* < .001 *, 95 % CI[.44, .68]	.39, *p* < .001 *, 95 % CI[.27, .53]	.39, *p* < .001 *, 95 % CI[.23, .53]
Trauma Load (Life Event Checklist)				
in Childhood	3.5 (2.7)	.43, *p* < .001 *, 95 % CI[.27, .57]	.22, *p* = .014 *, 95 % CI[.06, .38]	.30, *p* = .001 *, 95 % CI[.16, .46]
in Adulthood	2.0 (1.9)	.32, *p* < .001 *, 95 % CI[.12, .50]	.27, *p* = .002 *, 95 % CI[.08, .45]	.07, *p* = .431, 95 % CI[-.13, .25]
MACE Severity of Types				
Emotional Neglect	3.5 (2.4)	.43, *p* < .001 *, 95 % CI[.28, .56]	.37, *p* < .001 *, 95 % CI[.22, .51]	.32, *p* < .001 *, 95 % CI[.17, .46]
Non-verbal Emotional Abuse	3.7 (2.6)	.41, *p* < .001 *, 95 % CI[.27, .53]	.34, *p* < .001 *, 95 % CI[.07, .21]	.23, *p* = .010 *, 95 % CI[.07, .38]
Parental Verbal Abuse	5.1 (3.6)	.41, *p* < .001 *, 95 % CI[.27, .54]	.25, *p* = .005 *, 95 % CI[.08, .40]	.29, *p* = .001 *, 95 % CI[.12, .44]
Peer Emotional Abuse	5.9 (3.1)	.29, *p* = .001 *, 95 % CI[.13, .42]	.33, *p* < .001 *, 95 % CI[.19, .46]	.16, *p* = .064, 95 % CI[.00, .33]
Peer Physical Bullying	3.4 (2.7)	.30, *p* = .001 *, 95 % CI[.12, .46]	.14, *p* = .106, 95 % CI[.01, .28]	.17, *p* = .057, 95 % CI[-.01, .32]
Parental Physical Maltreatment	4.0 (2.8)	.28, *p* = .001 *, 95 % CI[.09, .46]	.14, *p* = .174, 95 % CI[-.05, .33]	.22, *p* = .014 *, 95 % CI[.05, .38]
Physical Neglect	1.6 (1.9)	.43, *p* < .001 *, 95 % CI[.27, .58]	.21, *p* = .017 *, 95 % CI[.03, .38]	.19, *p* = .033 *, 95 % CI[.01, .35]
Sexual Abuse	0.9 (1.5)	.51, *p* < .001 *, 95 % CI[.35, .65]	.23, *p* = .008 *, 95 % CI[.08, .41]	.26, *p* = .003 *, 95 % CI[.09, .40]
Witnessing Interparental Violence	1.3 (2.4)	.22, *p* = .013 *, 95 % CI[.02, .41]	.10, *p* = .268, 95 % CI[-.08, .29]	.22, *p* = .014 *, 95 % CI[.06, .39]
Witnessing Violence to Siblings	2.2 (2.9)	.35, *p* < .001 *, 95 % CI[.15, .52]	.22, *p* = .013 *, 95 % CI[.05, .37]	.29, *p* = .001 *, 95 % CI[.09, .46]

* Statistically significant at the adjusted alpha level using the Benjamini-Hochberg correction procedure

The trauma load in childhood measured with the event Checklist was related to all three symptom dimensions (for PTSD symptoms *r* = .43, *p* < .001, 95 % CI[.27, .57], for symptoms of shutdown dissociation *r* = .22, *p* = .014, 95 % CI[.06, .38] and for symptoms of depression *r* = .30, *p* = .001, 95 % CI[.16, .46]. In addition, the trauma load in adulthood was related to symptoms of PTSD *r* = .32, *p* < .001, 95 % CI[.12, .50] and shutdown dissociation *r* = .27, *p* = .002, 95 % CI[.08, .45], but not to symptoms of depression *r* = .07, *p* = .431, 95 % CI[-.13, .26].

### Modeling severity of symptoms of PTSD, shutdown dissociation and depression (Hypothesis 2)

#### Modeling symptoms of PTSD

**Fig. 3:**
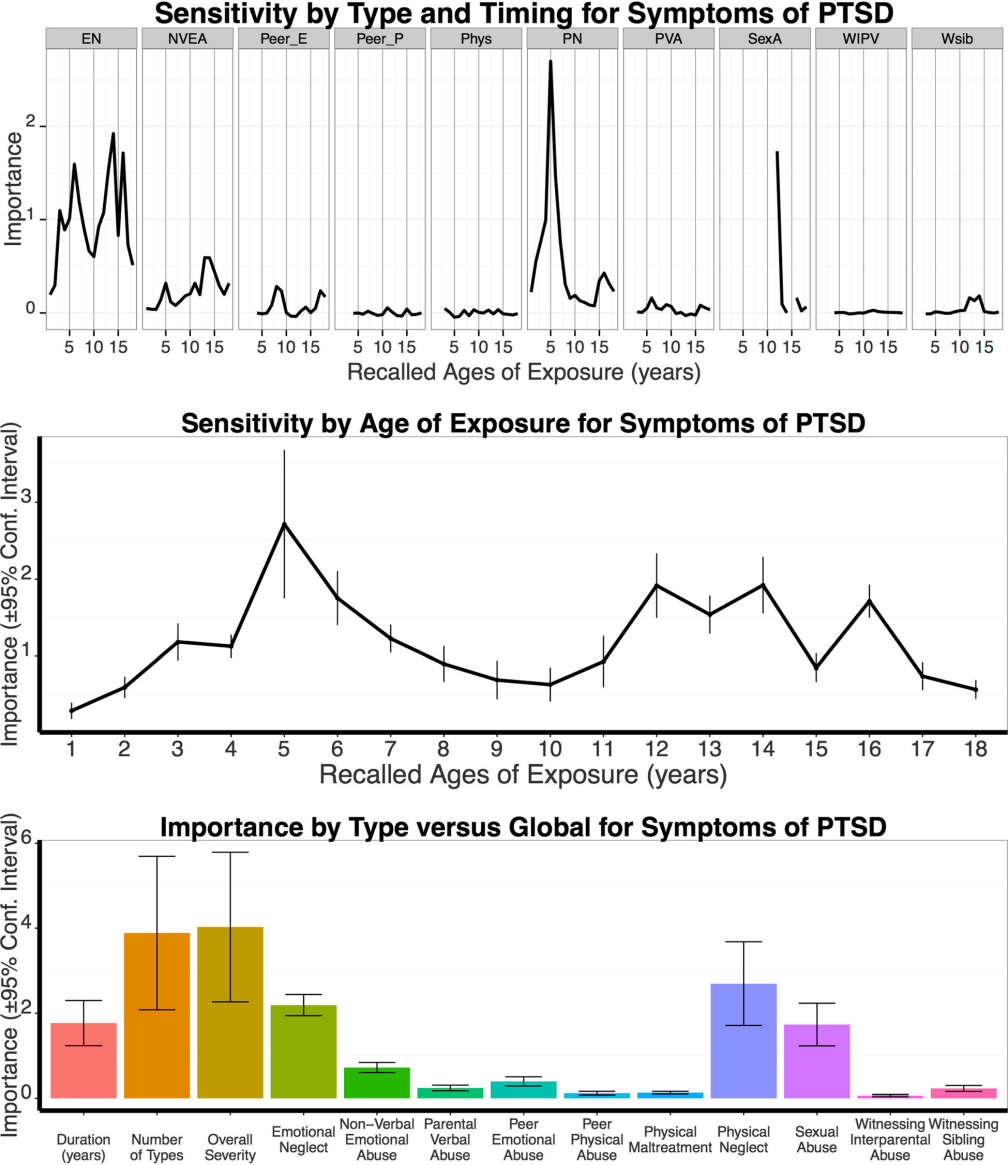
Results from Random Forest Regressions Modeling Symptoms of PTSD. Top: Sensitivity by Type and Timing Results. Importance of type is represented per age (abscissa: years between 1-18) for the MACE types EN: emotional neglect, NVEA: non-verbal emotional abuse, Peer_E: peer emotional abuse, Peer_P: peer physical bullying, Phys: parental physical maltreatment, PN: physical neglect, PVA: parental verbal abuse, SexA: sexual abuse, WIPV: witnessing interparental violence, Wsib: witnessing violence to siblings. Mid: Sensitive Period regardless of Type. Bottom: Type versus Global Model. Note: For the sensitivity of type and timing analysis (results displayed in A) missing values point to specific types and timings with low reported exposure < 5 %. Values ≤ 0 have no predictive importance

Within the ‘Sensitivity by Type and Timing’ model conditioned random forest regression verified that exposure to physical neglect at the age of 5 was most pronounced for PTSD symptoms (Fig. 3, upper graph; predictive importance *M* = 2.70, *SD* = 1.40). Furthermore, emotional neglect at ages 6, 14 and 16, sexual abuse at age 12, and non-verbal emotional abuse at age 14 showed enhanced predictive strength for PTSD symptoms, resulting in the biphasic sensitivity pattern with peak vulnerability at age 5–6 and 12–16 (Fig. 3).

Contrasting the relative importance obtained by maximal peak of physical neglect at age 5 with the predictive strength of the global measure of MACE duration (*M* = 1.78, *SD* = 0.76) showed a significant difference (*t*_9_ = 3.76, *p* = .004). Yet, addressing the multiplicity model the PTSD symptom severity was better explained by the number of different adverse experiences (*M* = 3.89, *SD* = 2.56) than by physical neglect at age 5 (*t*_9_ = -2.48, *p* = .034). Furthermore, MACE overall severity (*M* = 4.03, *SD* = 2.50) was more important in predicting PTSD symptoms than the type and timing specific peak (*t*_9_ = -3.14, *p* = .012). When considering physical neglect for a 2-year window (age 5–6, importance *M* = 4.16, *SD* = 1.95), the predictive strength of physical neglect at ages 5–6 for PTSD symptoms was not better than the predictive strength of MACE overall severity (*t*_9_ = 0.34, *p* = .721) or multiplicity (*t*_9_ = 0.62, *p* = .552).

No better prediction of PTSD symptoms was achieved by trauma load in adulthood (*M* = 0.17, *SD* = 0.12), trauma load in childhood (*M* = 1.09, *SD* = 0.29), gender (*M* = 0.20, *SD* = 0.12), and diagnostic categories F1 (no importance), F2 (*M* = 0.78, *SD* = 0.42) and F6 (*M* = 0.17, *SD* = 0.12), neuroleptic medication (*M* = 0.03, *SD* = 0.10), antidepressant medication (*M* = 0.09, *SD* = 0.08), as well as number of hospitalization (*M* = 0.03, *SD* = 0.06; importance and its standard deviation in brackets). The best predictors of PTSD symptom severity derived from the Forest Regression analysis were MACE overall severity, followed by physical neglect at age 5–6 as well as MACE multiplicity.

Post-hoc analyses using linear modeling unveiled how the measures (overall severity, physical neglect at age 5, sexual abuse at age 12, emotional neglect at age 6 and 14) may have fostered PTSD symptoms. According to the AIC, MACE overall severity, physical neglect at age 5, sexual abuse at age 12 and emotional neglect at age 6 gained importance in a linear model explaining 46.5 % of the variation of PTSD symptoms (see Table 3A for regression coefficients). The residuals of the model did not deviate from normality (*Z* = 1.27, *p* = .080).

**Table 3:** Results of the linear Regression for Variables Predicting (A) PTSD Symptoms, (B) Shutdown Dissociation and (C) Depression

Model	Unstandardized Coefficients *B*	Unstandardized Coefficients *SE*	Standardized Coefficient *ß*	*t*	*p*
A. Results of the linear Regression for Variables Predicting PTSD Symptoms					
Constant	−3.28	1.75		−1.88	*p* = .063
MACE SUM	0.21	0.06	.29	3.42	*p* = .001
Physical Neglect at age 5	2.28	0.80	.24	3.86	*p* = .005
Emotional Neglect at age 6	0.60	0.43	.12	1.41	*p* = .161
Sexual Abuse at age 12	6.73	1.89	.25	3.56	*p* = .001
B. Results of the linear Regression for Variables Predicting Symptoms of Shutdown Dissociation					
Constant	3.47	0.73		−1.88	*p* < .001
Physical Neglect at age 5	0.70	0.48	.14	1.46	*p* = .147
Emotional Neglect at age 4	0.57	0.26	.21	2.17	*p* = .032
Parental Non-verbal Emotional Abuse at age 14	0.44	0.26	.16	1.73	*p* = .086
Sexual Abuse at age 12	3.02	1.12	.22	2.69	*p* = .008
C. Results of the linear Regression for Variables Predicting Symptoms of Depression					
Constant	14.82	1.29		11.48	*p* < .001
F2 diagnosis	−5.12	0.14	−.34	−4.49	*p* < .001
MACE duration	0.01	0.01	.15	1.39	*p* = .166
Emotional Neglect at age 9	0.51	0.30	.17	1.67	*p* = .097
Sexual Abuse at age 12	2.69	1.37	.16	1.97	*p* = .051

#### Modeling symptoms of shutdown dissociation

**Fig. 4:**
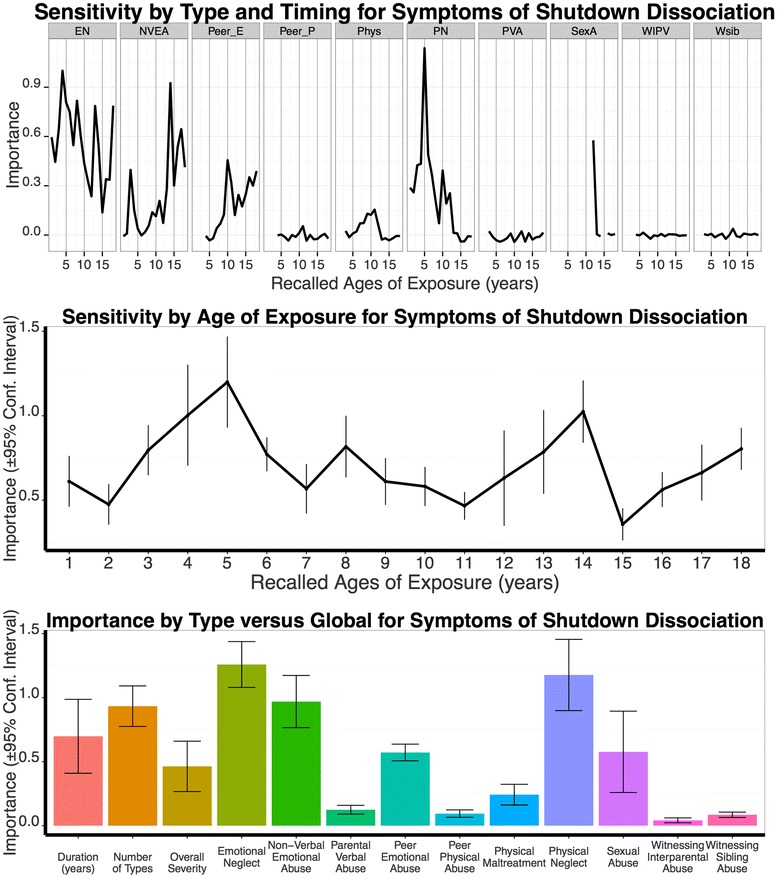
Results from Random Forest Regressions Modeling Shutdown Dissociation. Same legend as in Fig. 3

The ‘Sensitivity by Type and Timing’ analysis showed the highest importance for physical neglect at age 5 (*M* = 1.14, *SD* = 0.44; Fig. 4). Moreover, emotional neglect at ages 4 to 6, 8 and 13 obtained predictive strength for shutdown dissociation. Non-verbal emotional abuse was proposed as a third relevant type, in particular at age 14, while sexual abuse at age 12 contributed to the sensitivity for shutdown dissociation as well. Taken together, for symptoms of dissociation the ‘Sensitivity of Type and Timing’ model resulted in a broad susceptibility to emotional neglect and to two major sensitive periods peaking at ages 3–6 and 12–14 (compare Fig. 4).

Comparing the importance of the MACE global scores duration (*M* = 0.70, *SD* = 0.22) and overall severity (*M* = 0.46, *SD* = 0.28) to the highest single peak of the ‘Sensitivity by Type and Timing’ indicated better prediction for physical neglect at age 5 than MACE duration (*t*_9_ = 2.25, *p* = .050) and MACE overall severity (*t*_9_ = 3.40, *p* = .008), whereas physical neglect at age 5 did not predict shutdown dissociation better than the MACE multiplicity (*M* = 0.93; *SD* = 0.22), *t*_9_ = 1.69, *p* = .125. Emotional neglect showed the highest peak when two adjacent years (age 4–5) were considered. The predictive strength of emotional neglect at age 4–5 was higher (*M* = 1.81, *SD* = 0.62) than the predictive strength of each global MACE measures: duration, *t*_9_ = 4.73, *p* = .001, multiplicity, *t*_9_ = 4.51, *p* = .001, and overall severity, *t*_9_ = 5.46, *p* < .001. In contrast, the predictors trauma load in adulthood (*M* = 0.21, *SD* = 0.19), traumatic experiences in childhood (*M* = 0.13, *SD* = 0.13), gender (*M* = 0.21, *SD* = 0.13), F1 diagnoses (*M* = 0.07, *SD* = 0.09), F2 diagnoses (*M* = 0.23, *SD* = 0.19), F6 diagnoses (no importance), neuroleptic medication (*M* = 0.05, *SD* = 0.11), antidepressant medication (no importance), and number of hospitalizations (*M* = 0.06, *SD* = 0.14) did not predict dissociative symptoms better than the ‘Sensitivity by Type and Timing’ model (importance and its standard deviation in brackets).

Linear model selection was accomplished for the predictors derived from the ‘Sensitivity by Type and Timing’ analysis: MACE multiplicity, physical neglect at age 5, emotional neglect at age 4, parental non-verbal emotional abuse at age 14, and sexual abuse at age 12. According to the AIC, the best fitting model included the variables emotional neglect at age 4, physical neglect at age 5, and sexual abuse at age 12 and parental non-verbal emotional abuse at age 14. This model explained altogether 26.7 % of the variation of shutdown dissociation symptoms (Table 3B). The residuals of the model did not deviate from normality (*Z* = 0.87, *p* = .431).

#### Modeling symptoms of depression

**Fig. 5:**
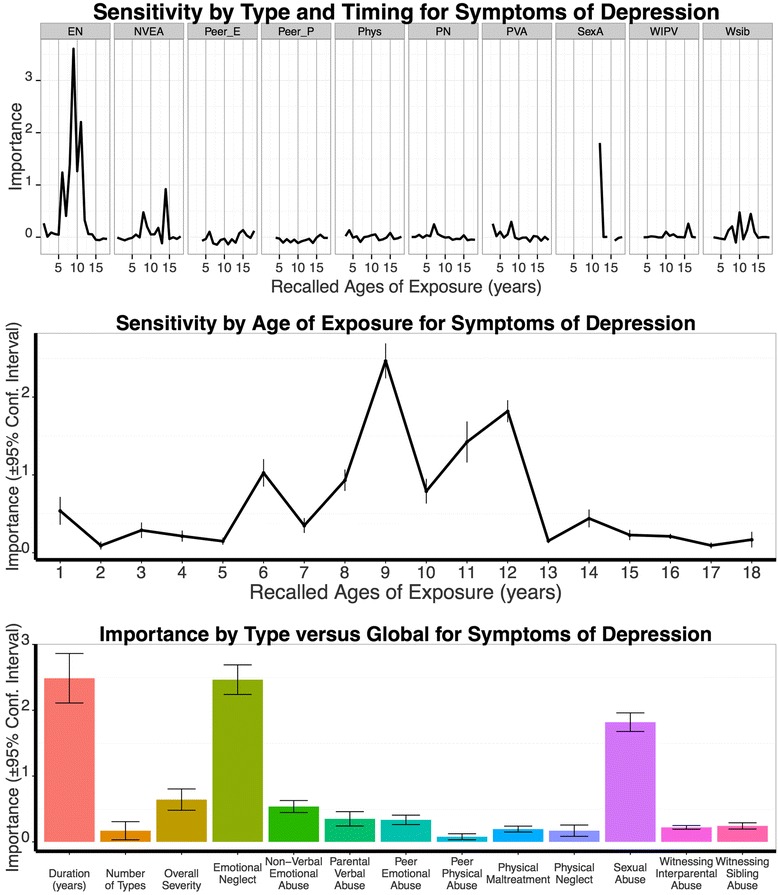
Results from Random Forest Regressions Modeling Depression Symptoms. Same legend as in Fig. 3

The ‘Sensitivity by Type and Timing’ model indicated the highest predictive strength for emotional neglect at age 9 (importance *M* = 2.46, *SD* = 0.32). Furthermore, type and timing specific sensitivity was suggested for sexual abuse at age 12 and parental non-verbal emotional abuse at age 14 (Fig. 5).

The peak of the ‘Sensitivity by Type and Timing’ model, emotional neglect at age 9, predicted symptoms of depression better than MACE multiplicity (*M* = 0.17, *SD* = 0.20; *t*_9_ = 21.72, *p* < .001) and MACE overall severity (*M* = 0.64, *SD* = 0.23; *t*_9_ = 12.60, *p* < .001), but not better than MACE duration (*M* = 2.49, *SD* = 0.53; *t*_9_ = -0.16 *p* = .879). Predictive power was better for emotional neglect at the age window 8-9 (*M* = 3.38, *SD* = 0.30) than MACE duration (*t*_9_ = 5.06, *p* < .001).

In addition, meeting a psychotic spectrum diagnosis (importance *M* = 13.51, *SD* = 1.39) and neuroleptic medication (*M* = 8.03, *SD* = 0.86) varied severity of depression. Patients with a schizophrenic spectrum disorder had lower depression scores (*M* = 12.1, *SD* = 6.6) than non-psychotic patients *M* = 19.3 (*SD* = 6.4). All other predictors did not show better predictive importance for symptoms of depression: trauma load in adulthood (no importance), trauma load in childhood (*M* = 0.54, *SD* = 0.17), gender (*M* = 0.38, *SD* = 0.17), medication with antidepressants (*M* = 0.96, *SD* = 0.38), diagnostic categories F1 and F6 as well as number of hospitalizations (no importance).

Based on the ‘Sensitivity of Type and Timing Analysis’, F2 diagnoses, MACE duration, emotional neglect at age 9, parental non-verbal emotional abuse at age 14, and sexual abuse at age 12 were included as variables in the linear regression. Using AIC, the best-fitting model combined a negative impact of the binary variable psychotic spectrum disorder, indicating lower depression scores for patients with psychotic spectrum disorder, and positive regression coefficients for MACE duration, emotional neglect at age 9 and sexual abuse at age 12. The overall model obtained an adjusted *R*^*2*^ of 0.34 (compare Table 3C for regression coefficients). The residuals of the model did not deviate from normality (*Z* = 0.74, *p* = .649).

## Discussion

The present study examined how a detailed analysis of type and timing of childhood adversities complements the understanding of sensitive periods for symptoms frequently reported in patients with psychiatric disorders. Depression, dissociation, and PTSD symptoms were selected as prominent in patients diagnosed with stress-related, affective, and personality disorders, but also as frequently reported comorbid symptoms in other diagnostic groups, including substance abuse and psychotic disorders e.g., [9–11]. Moreover, recruiting a larger transdiagnostic sample targeted the nature of ACE effects on severity of mental illness, as indicated by symptom severities. Comparing potential effects of experience load (or ‘dose’ effect) with those of certain experiences at specific, sensitive periods during development might suggest a common role of environmental stress in the development of symptoms, independent of disorder specific factors. The present study sought to clarify the impact of adversities on the course and severity of illness. Two hypotheses on the nature of this influence were tested: (1) stressful/traumatic experiences unfold psychopathology in vulnerable individuals in a dose-dependent manner, and (2) stressful/traumatic experiences are influential particularly at a certain time, i.e., during sensitive developmental periods. Referring to the intensity of common symptoms, both types of influence should be valid across diagnoses.

In line with previous findings the present results confirm dose-dependent effects of ACE (multiplicity and overall severity) and traumatic experiences on severity of all three dimensions of symptoms [3, 6]. Moreover, the association between the MACE duration score and symptom severity scores suggests a common effect of elevated stress ‘load’: the earlier the abuse and neglect occur and the longer they last, the more severe the indices of psychopathology in adult patients. However, the effects of ACE type and timing on the severity of each symptom dimension indicate that certain types of experiences at specific sensitive periods have stronger impact on and more predictive strength for symptom development than global stress load (MACE measures duration, multiplicity and overall severity). Regarding type of experience, the present results emphasize the importance of emotional and physical neglect, which may have been underestimated when focusing exclusively on sexual abuse and traumata. Regarding timing of experience, the present results emphasize sensitive developmental periods between age 5 and 14, thus preschool age and early adolescence. The fine-grained analysis of windows of vulnerability for type and timing of ACE strengthens the model of sensitive periods of brain development [7, 8]. Periods around age 3–5 have been associated with hippocampus development [7], and may therefore be particularly sensitive for vulnerability for ACE and traumata fostering later dissociation and PTSD. Further windows of vulnerability affecting hippocampal development have been defined for ages 11–13, and windows affecting amygdala development (age 10–11) and the prefrontal cortex (ages 14–16) [7, 8].

The similar type and timing profile suggests a shared vulnerability of ACE to foster the co-occurrence of dissociation and PTSD symptoms e.g., [46]. The overlapping vulnerability for both symptom dimensions highlighted physical and emotional neglect experiences around pre-school ages as well as adolescent sexual abuse, resulting in biphasic susceptibility to ACE. Similar timings have been highlighted in the literature for symptoms of PTSD, showing enhanced vulnerability for the earlier [20], and also for the later window of higher vulnerability [21]. Again, physical and emotional neglect as well as sexual abuse emerged as important predictors for shutdown dissociation [15, 25], while also complementing prior reports in showing more pronounced vulnerability for preschool and adolescent adversities. In line with previous findings on symptoms of depression that highlighted ACE effects before age 12 [21, 29, 30], the current results demonstrate enhanced vulnerability for emotional neglect at school ages 6–11. Even though we observed a type specific peak of parental non-verbal abuse at age 14 for depressive symptoms, which is in line with the results of Khan and colleagues [31], this was not the most important predictor. The present results may be affected by sample selection. The present results are confined to patients who have developed mental illness later in life and all report at least some symptoms of depression, whereas the community sample consisted of individuals from sub threshold to clinical levels of psychopathology as well as lifetime depression. Considering the length of the sensitive period, current results suggest relevant sensitive periods of at least two consecutive years of ACE exposure for stress-related symptoms, which is also in line with neurobiological findings [7, 8]. By supporting these periods of vulnerability and drawing attention to the particular sensitive for emotional and physical neglect, the present results encourage further research on the neuroendocrine mechanisms that might mediate ACE effects on brain function and structure [7, 8], hypothalamic-pituitary-adrenal-axis regulation [47] or epigenetic profiles [48], finally resulting in enhanced vulnerability for psychopathological developments.

Limitations of the present study have to be considered: First, retrospective assessment of ACE prompted concerns regarding the veracity and reliability of retrospective, memory-based subjective ratings, in patients with severe mental illness and lower levels of cognitive functioning. However, a review by Brewin and colleagues indicated scant evidence in support of these concerns [49]. Excellent retest-reliability has been provided for the MACE scale [14], and other studies demonstrate sufficient test-retest reliability for comparable instruments of retrospective assessment of ACE in severe mental illness [50, 51]. Reports appeared to be highly consistent over years and did not significantly vary with the severity of psychotic or depressive symptom [52]. In contrast to the false positive bias, there has been support for underreporting in retrospective assessment [53–55]. Besides this bias of false negatives, the reported ACE did not obtain enough positive ratings at early ages, even though their negative impact on the development has been demonstrated [56]. Abuse recollection might be limited before age 3 [57]. For the present study, e.g. physical abuse before the age of 3, sexual abuse before the age of 12, witnessed interparental violence and violence towards siblings as well as peer emotional and physical before the age of 3 was not considered in the forest regression models. Second, we cannot completely rule out effects of medication on symptoms of PTSD, shutdown dissociation and depression, however a major impact is unlikely as demonstrated by the predictive importance values as well as stability of peak types and timings for each symptom dimension (with and without the consideration of the medication). Third, the present results are constraint to a clinical sample of psychiatric patients and, thus, have limited generalizability. A cross-validation of the hypotheses testing in a community sample would certainly be necessary before drawing any conclusion to the general population or prevention. However, a comparison of inpatients and a symptom-free community sample would direct the attention to the relationship of stress/trauma on the emergence of psychopathology (per se), rather than on the expression of psychopathology in symptom severity—as emphasized in the present study.

## Conclusion

The present study confirmed the positive association of childhood adversities to psychopathology. In particular the results emphasize the impact of physical and emotional neglect besides sexual abuse. Regarding the timing, pre-school age (4–6) and pre-adolescent (8–9) seemed to be more vulnerable periods. PTSD, the most severe stress-related disorder, clearly varies with the amount of adverse experiences irrespective of age of experience supporting the dose-dependent model. For symptoms of dissociation and depression, the present results provided evidence for the sensitive period model. The specific pattern of multiplicity, overall severity as well as type and timing specific effect provides evidence for adversity-related processes that are present in a sample with low levels of hospitalization and young age. Symptoms of depression and anxiety are often considered as unspecific, however might provide a framework to prevent further negative outcomes that are related to childhood adversities such chronic illness courses [43, 58–60]. It is crucial to understand the negative impact of specific effects of adversities and trauma on the psychopathology to advance the models of vulnerability and learn how to adapt treatment strategies to counterstrike adverse outcomes.

## Footnotes

1 The spectrum of F5 diagnosis was excluded from the analysis due to low frequency (3.2 %). We further refrained to consider the F4 since there is a high dependency with reported symptoms for PTSD and dissociation.
